# Infection of nonclassic monocytes by respiratory syncytial virus induces an imbalance in the CD4^+^ T-cell subset response

**DOI:** 10.1128/spectrum.02073-24

**Published:** 2024-12-10

**Authors:** Lianlian Han, Danyang Li, Conghui Wang, Lili Ren, Li Guo, Jianwei Wang

**Affiliations:** 1 NHC Key Laboratory of Systems Biology of Pathogens and Christophe Mérieux Laboratory, National Institute of Pathogen Biology, Chinese Academy of Medical Sciences & Peking Union Medical College, Beijing, China; 2 Key Laboratory of Pathogen Infection Prevention and Control (Ministry of Education), State Key Laboratory of Respiratory Health and Multimorbidity, National Institute of Pathogen Biology, Chinese Academy of Medical Sciences & Peking Union Medical College, Beijing, China; 3 Key Laboratory of Respiratory Disease Pathogenomics, Chinese Academy of Medical Sciences & Peking Union Medical College, Beijing, China; Shandong First Medical University, Jinan, Shandong, China

**Keywords:** RSV, nonclassic monocyte, Treg, Th2, IL-10, IL-1β, TNF-α

## Abstract

Respiratory syncytial virus (RSV) causes lower respiratory tract infections in infants and young children, leading to a pathogenesis-associated imbalance in CD4^+^ T-cell subsets and monocyte subsets. To investigate whether RSV affects the imbalance of CD4^+^ T-cells through monocytes, we examined the effects of the RSV-infected monocyte subset on CD4^+^ T-cell subsets, namely, Th1, Th2, Th17, and regulatory T (Treg) subsets, on proliferation *in vitro* and identified key monocyte-derived cytokines. We found that RSV efficiently infects CD16^+^ monocytes, but not CD16^-^ monocytes, *via* cocultures of CD4^+^ T-cells with RSV-infected CD16^+^ monocytes, inhibits Treg cell proliferation and increases Th2 cell frequency, suggesting that RSV causes an imbalance in the CD4^+^ T-cell subset by primarily infecting CD16^+^ monocytes. Our data also revealed that IL-1β and IL-10 are key cytokines responsible for the activities of RSV-infected CD16^+^ monocytes. In a mouse model, we found that high-efficiency RSV infection of mouse Ly6C^-^ monocytes, corresponding to CD16^+^ monocytes in humans, and adoptive transfer of Ly6C^-^ monocytes during RSV infection decreased the Treg frequency in the lungs and aggravated pneumonia. Our data indicate that RSV can increase its pathogenesis through infection of nonclassic monocytes, leading to a CD4^+^ T-cell imbalance.

**IMPORTANCE:**

This study identified a pathogenesis pathway related to the RSV-nonclassic monocyte–IL-1β/IL-10-CD4^+^ T-cell subset balance, which links the heterogeneity of monocytes to RSV pathogenesis and elucidates a new mechanism by which RSV infection disrupts the balance of CD4^+^ T cells during RSV infection. These new findings provide potential therapeutic targets for RSV infection.

## INTRODUCTION

Respiratory syncytial virus (RSV) is, globally, a major, viral etiological agent of acute respiratory tract infections (ARTIs). It can cause significant morbidity and hospitalizations in young children, immunodeficient adults, and elderly individuals. RSV infections caused an estimated 33.0 million episodes, 3.6 million hospitalizations, and 26,300 in-hospital deaths in children aged 0–60 months worldwide in 2019 (1), resulting in a substantial burden on health-care services. In total, RSV-ARTI leads to an approximately 45% hospitalization rate in children younger than 6 months (2). However, our understanding of RSV pathogenesis and the host immune response is incomplete (3–5).

RSV infection induces a host inflammatory response, which is indispensable for viral clearance but also leads to lung damage (6, 7). Previous studies have indicated that the CD4^+^ T-cell response contributes to this immune-mediated pathology (8). CD4^+^ T-cells constitute diverse subsets with distinct immune functions, including Th1, Th2, Th17, and regulatory T (Treg) cells (9, 10). RSV infection can significantly affect the frequency and activity of distinct CD4^+^ T-cell subsets and cause an imbalance in the CD4^+^ T-cell immune response, which in turn contributes substantially to disease severity (11). For example, a biased Th2 response is associated with more severe RSV disease in patients, and RSV clearance is delayed in mice in which IL-4 is overexpressed (12). In RSV-infected patients, the number of Tregs, an immune inhibitory cell subset, is decreased (13). Treg depletion in mice also leads to a greater inflammatory response to RSV infection and a delay in RSV clearance. However, although an improper CD4^+^ T-cell response can exacerbate RSV disease, the underlying mechanism by which RSV modulates CD4^+^ T-cell subsets is poorly understood.

Monocytes, innate immune cells, quickly respond to infection and modulate the subsequent immune reaction. Monocytes are heterogeneous cell populations that can be divided into CD16^-^ monocytes (classic monocytes, CMo) and CD16^+^ monocytes in humans, corresponding to Ly6C^+^ and Ly6C^-^ monocytes in mice. In humans, the CD16^+^ subset can be further classified into intermediate monocyte (CD14^high^CD16^+^) and nonclassic monocyte (NCMo, CD14^dim^CD16^+^) subsets (14). Monocyte subsets exhibit distinct innate immune responses to pathogens. Classic monocytes respond primarily to TLR4 (15), whereas nonclassic monocytes are sensitive to TLR7/8 simulation but insensitive to TLR4 agonists during infection (16). In addition to innate immune responses, monocyte subsets also demonstrate distinct activities in regulating adaptive immune reactions, including CD4^+^ T-cell subsets. For example, CD16^+^ monocytes increase the numbers of Th1 cells and decrease the numbers of Treg cells through IL-12 signaling (17), but CD16^-^ monocytes inhibit Helios^-^ Tregs through tumor necrosis factor-alpha (TNF-α) signaling (18). However, the exact interactions regarding the heterogenicity of the innate immune system and the ability to modulate CD4^+^ T-cell immune responses have not been studied in detail for RSV-associated disease.

Previous studies have shown that human monocytes can be infected with RSV *in vitro* (19). In addition, RSV infection can modulate immune cell costimulatory molecules, including CD80, CD86, CD40, and HLA-DR, which are expressed in monocytes. RSV infection can also stimulate the production of multiple cytokines and chemokines by monocytes (20). These costimulatory molecules, cytokines, and chemokines can regulate CD4^+^ T-cell immune responses. Although the frequency of CD14^+^CD16^+^ cells is increased in RSV patients and is associated with disease severity (21), the susceptibility and innate response of distinct monocyte subsets to RSV infection remain unclear. Whether RSV increases viral pathogenicity by skewing the CD4^+^ T-cell immune response through infection of distinct monocyte subsets needs to be determined.

In the present study, we assessed the effects of RSV-infected human monocytes and monocyte subsets on the frequency of distinct human CD4^+^ T-cell subsets *in vitro* and identified the key cytokines responsible for monocyte activity. Using RSV mouse models and adoptive monocyte subset transfer, we also analyzed the susceptibility of monocyte subsets to RSV infection *in vivo* and identified the role of monocyte subsets in RSV pathogenicity and CD4^+^ T-cell subset polarization *in vivo*.

## RESULTS

### Human monocytes infected with RSV inhibit Treg cell proliferation but promote Th2 cell proliferation

**Fig 1:**
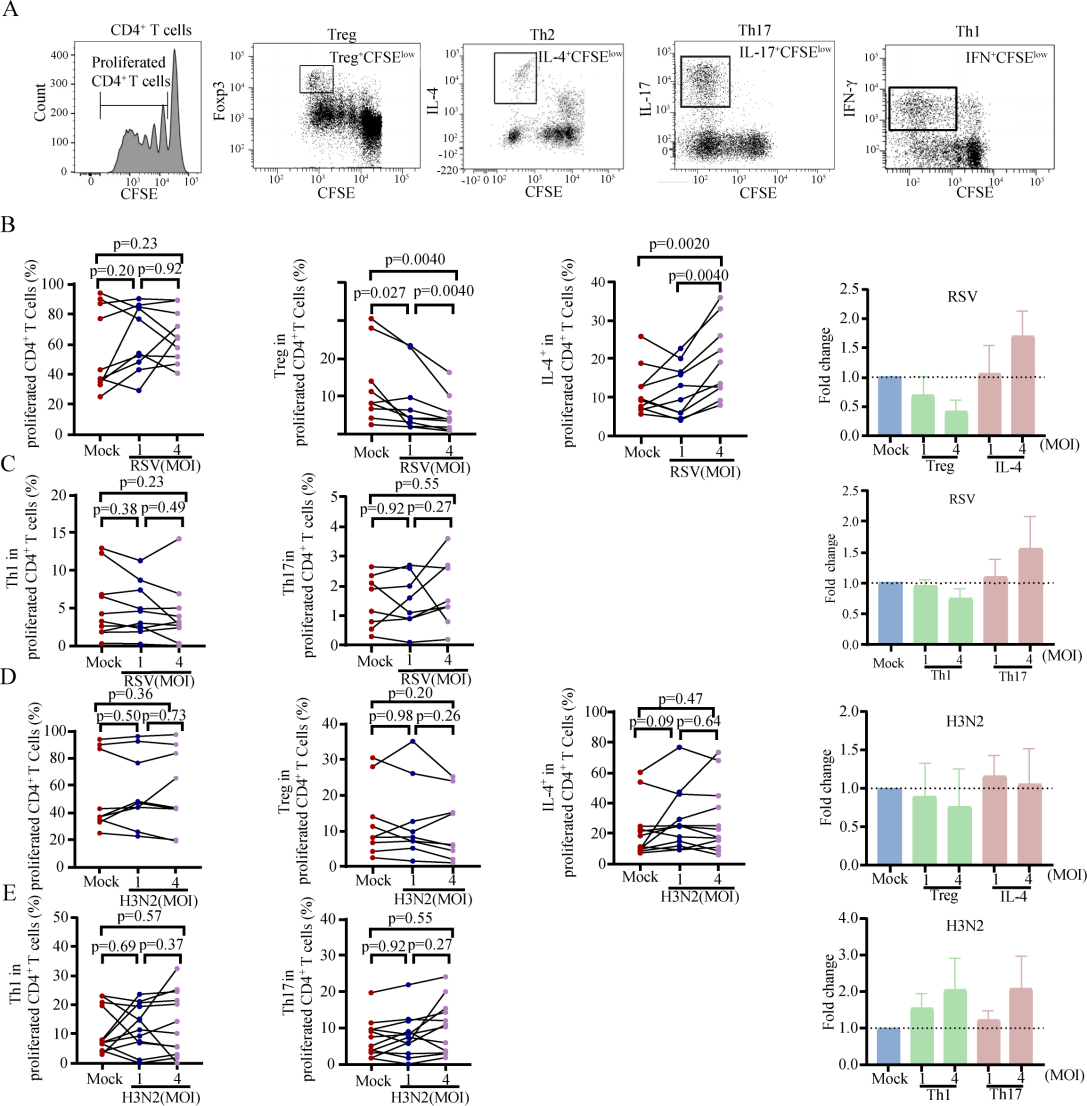
RSV-infected monocytes inhibit Treg cell expansion but promote Th2 cell proliferation. Monocytes with or without RSV or influenza A virus H3N2 infection were cocultured with total CFSE-stained CD4^+^ T-cells for 7 days. Monocyte without RSV or H3N2 infection were as mock group. In coculture, both the quantity of monocytes and CD4^+^ T cells was measured at 2.5 × 10^4^. The proliferation of CD4^+^ T-cells and Th1, Th2, Th17, and Treg cells was analyzed by flow cytometry. (**A**) The gating strategy for proliferating CD4^+^, Treg, Th1, Th2, and Th17 cells. (**B**) Changes in total CD4^+^ T-cell, Treg, and Th2 cell proliferation after coculture with RSV-infected monocytes. (**C**) Changes in Th1 and Th17 cell proliferation after coculture with RSV-infected monocytes. (**D**) Changes in total CD4^+^ T cell, Treg, and Th2 cell proliferation after coculture with H3N2-infected monocytes. (**E**) Changes in Th1 and Th17 cell proliferation after coculture with H3N2-infected monocytes. Multiple paired comparisons of cell proliferation changes were performed using the Friedman test.

To identify whether RSV infection skews the CD4^+^ T-cell subset balance by infecting monocytes, we tested the impact of human monocytes infected with RSV on CD4^+^ T-cell subset proliferation *in vitro*. First, total human monocytes were isolated from healthy donor peripheral blood and infected with RSV at 4°C or 37°C. The results showed that RSV can adsorb (Fig. S1A) and infect (Fig. S1B) monocytes. CD4^+^ T-cells were isolated from the peripheral blood of healthy donors. CFSE-stained CD4^+^ T-cells were then cocultured with monocytes with or without RSV infection. The Th1, Th2, Th17, and Treg frequencies among the proliferated CD4^+^ T-cells were analyzed *via* flow cytometry (Fig. 1A). We found that monocytes infected with RSV significantly inhibited the proliferation of Tregs (*P* = 0.004, median fold change = 0.35; Fig. 1B) but promoted the proliferation of Th2 cells (*P* = 0.002, median fold change = 1.53; Fig. 1B). RSV-infected monocytes did not significantly affect the total CD4^+^ T-cell count (*P* = 0.23, median fold change = 1.18; Fig. 1B), the proliferation of Th1 (*P* = 0.23, median fold change = 0.87; Fig. 1C) and Th17 cells (*P* = 0.55, median fold change = 1.2; Fig. 1C). To eliminate the interference of nonspecific responses induced by viral infections, we infected monocytes with influenza A virus H3N2 (H3N2) (Fig. S2) and analyzed the impact of H3N2 infection of monocytes on the proliferation of CD4^+^ T-cell subsets. The results revealed that monocytes infected with H3N2 did not significantly alter the proliferation of total CD4^+^ T cells or the proliferation of the Th1/Th2/Th17/Treg subsets (Fig. 1D and E). Our data indicate that RSV-infected monocytes can manipulate the balance of CD4^+^ T-cell subset proliferation by inhibiting Treg proliferation and promoting Th2 proliferation. These findings suggest that the skewed CD4^+^ T-cell balance present during RSV-ARTI may be caused by RSV-infected monocytes.

### RSV modulates Treg and Th2 cell proliferation through infection of CD16^+^ monocytes

**Fig 2:**
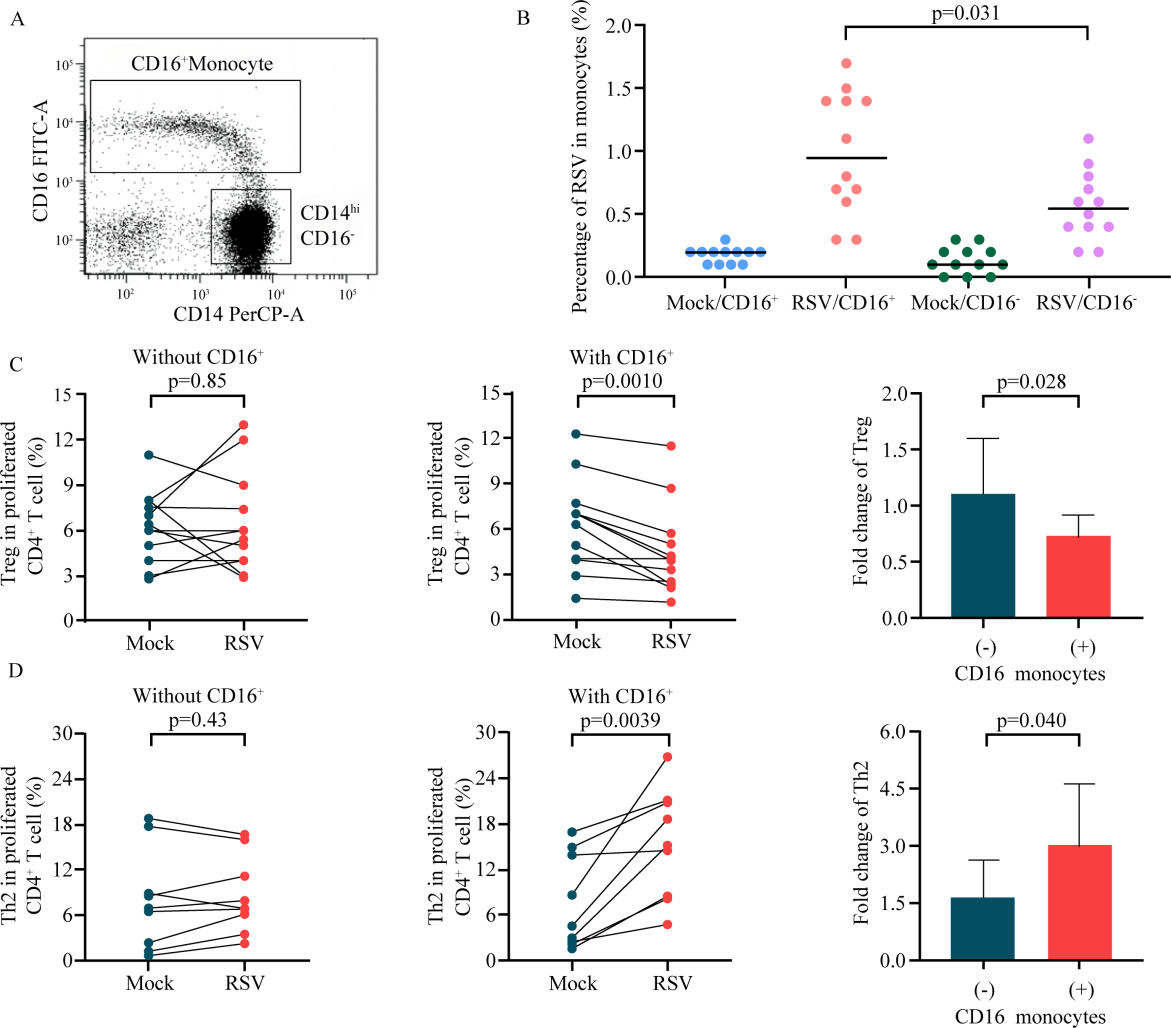
RSV-infected CD16^+^ monocytes modulate Treg and Th2 cell proliferation. (**A**) The gating strategy for CD16^+^ and CD16^-^ monocytes. (**B**) CD16^-^ and CD16^+^ monocyte subsets were sorted from PBMCs and cocultured with RSV. After 16 h, the frequency of RSV-infected cells was detected *via* intracellular staining with an APC-conjugated anti-RSV G protein antibody and analyzed *via* flow cytometry. (**C**) Treg proliferation was analyzed. Treg frequencies in the presence/absence of RSV-infected CD16^+^ monocytes are shown by pairs and fold changes. (**D**) Th2 proliferation was analyzed as described above. The frequency of Th2 cells in the presence/absence of RSV-infected CD16^+^ monocytes is shown by pairs and fold changes. Single comparisons between other metrics were performed *via* the Mann‒Whitney U test. Paired groups were compared with a two-tailed Wilcoxon matched-pairs signed-rank test.

Our data showed that the total number of RSV-infected monocytes can affect the number of CD4^+^ T-cell subsets and their respective balance. To elucidate the underlying mechanism, CD16^-^ and CD16^+^ monocyte subsets were isolated from PBMCs (Fig. 2A) and infected with RSV. RSV-infected cells were detected *via* intracellular staining with an anti-RSV G protein antibody. HEp-2 cells infected with RSV were used as positive controls (Fig. S3). Our results revealed that the percentage of RSV in CD16^+^ monocytes were higher than that in CD16^-^ monocytes (*P* = 0.031; Fig. 2B). These findings suggest that RSV mainly targets CD16^+^ monocytes during infection.

To ascertain whether RSV-infected CD16^+^ monocytes or CD16^-^ monocytes perturb the balance of CD4^+^ T-cell subsets, CD16^-^ and CD16^+^ monocytes were isolated from the peripheral blood of healthy donors and infected with RSV. Due to the limited quantity of CD16^+^ monocytes (only 5%–10% of the total monocytes), it is difficult to isolate the same number of CD16^+^ monocytes as the number of CD16^-^ monocytes for CD4^+^ T-cell/monocyte coculture experiments. Therefore, total CD4^+^ T cells were cocultured with CD16^-^ monocytes in the presence or absence of CD16^+^ monocytes. The proliferation of Th1/Th2/Th17/Treg cells was compared between cocultures of CD4^+^ T cells with CD16^-^ monocytes and those of CD4^+^ T cells with RSV-infected CD16^-^ monocytes or between cocultures of CD4^+^ T cells with CD16^-^ and CD16^+^ monocytes and those of CD4^+^ T cells with CD16^-^ and CD16^+^ monocytes infected with RSV. The results showed that RSV-infected CD16^-^ cells did not affect the proliferation of Tregs (*P* = 0.85; Fig. 2C) or Th2 cells (*P* = 0.43; Fig. 2D). However, RSV-infected CD16^+^ monocytes significantly suppressed the proliferation of Tregs (*P* = 0.0010; Fig. 2C) and enhanced the proliferation of Th2 cells (*P* = 0.0039; Fig. 2D). These data suggest that CD16^+^ monocytes constitute the crucial monocyte subset through which RSV influences the proliferation of Tregs and Th2 cells.

### RSV-infected CD16^+^ monocytes induce CD4^+^ T-cell subset imbalance through IL-10 and IL-1β signaling

**Fig 3:**
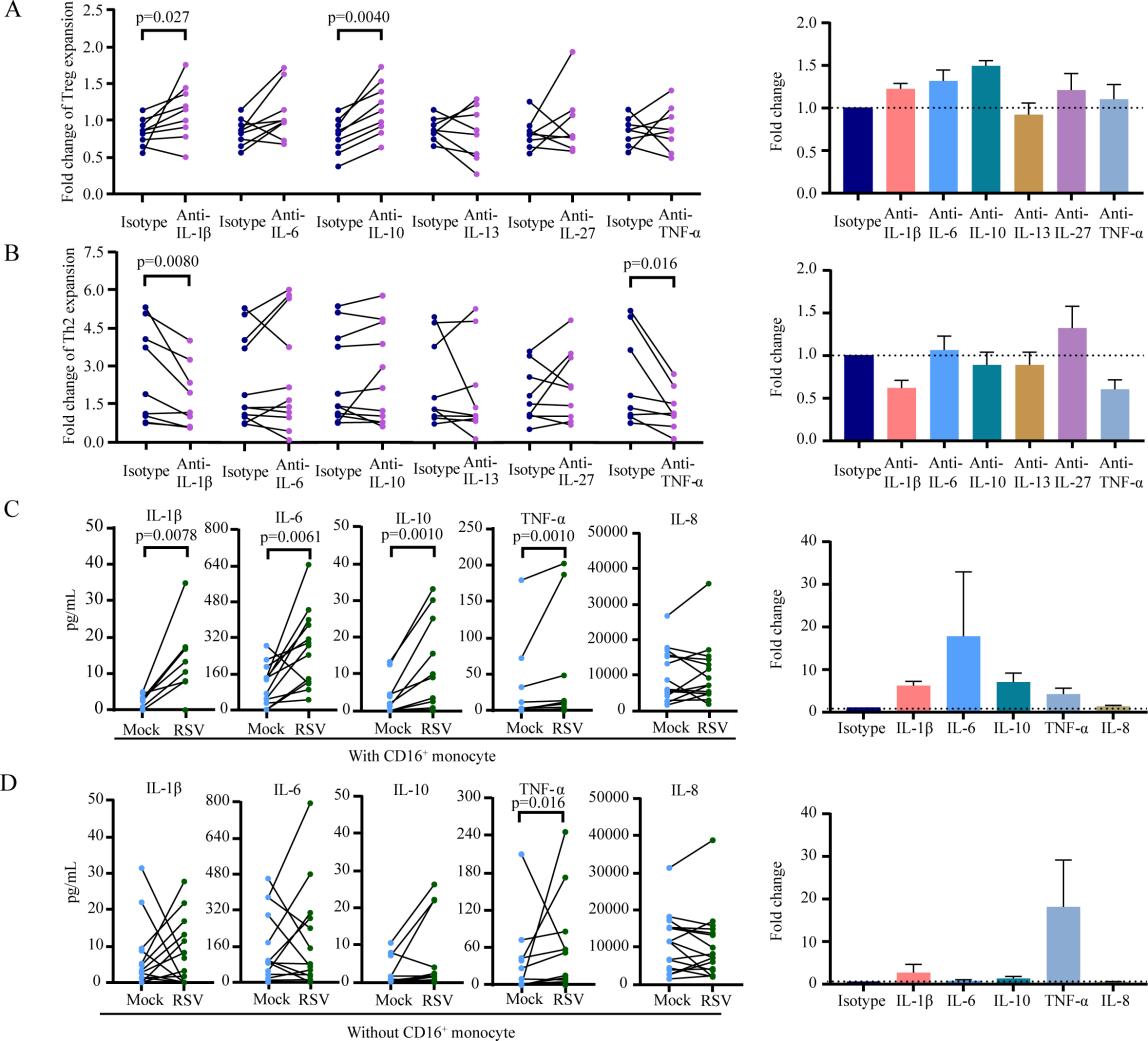
Cytokine effects on Treg and Th2 cell proliferation. RSV-infected monocytes and CD4^+^ T cells were cocultured. The effects of culture supernatant cytokines on Treg (**A**) and Th2 (**B**) cell proliferation were compared between the groups treated with and without neutralizing antibodies against cytokines. The cytokine levels in the culture supernatants of coculture with CD16^+^ monocytes (**C**) or without CD16^+^ monocytes (**D**) were measured *via* CBA. The number of CD4^+^ T-cells and CD16^-^ monocytes was measured at 2.5 × 10^4^, and the number of CD16^+^ monocytes was 1.25 × 10 ^4^ in the assay. Paired groups were compared with a two-tailed Wilcoxon matched-pairs signed-rank test.

Multiple monocyte-derived cytokines can regulate CD4^+^ T-cell subset polarization and activity, and their expression level can be altered by microbial infection (22–25). To identify the critical cytokines that disrupt the equilibrium of CD4^+^ T-cell subsets in RSV-infected CD16^+^ monocytes, we blocked the activities of various cytokines by using cytokine-specific neutralizing antibodies during the coculture of CD16^+^ monocytes and CD4^+^ T-cells. In the presence of control antibodies or cytokine-specific neutralizing antibodies, CD16^+^ monocytes infected with RSV were cocultured with total CD4^+^ T cells. The proliferation of Treg and Th2 cells was analyzed by flow cytometry, and the fold changes in the numbers of Treg and Th2 cells were calculated. Our data revealed that among the six candidate cytokines, namely, IL-1β, IL-6, IL-10, IL-13, IL-27, and TNF-α, neutralizing IL-1β (*P* = 0.027, fold change = 1.2; Fig. 3A) and IL-10 (*P* = 0.0040, fold change = 1.5; Fig. 3A) reversed the effects of RSV-infected CD16^+^ monocytes on Treg proliferation. In addition, the effects of RSV-infected CD16^+^ monocytes on Th2 cells were reversed by IL-1β (*P* = 0.0080, fold change = 0.62; Fig. 3B) and TNF-α blockade (*P* = 0.016, fold change = 0.61; Fig. 3B).

**Fig 4:**
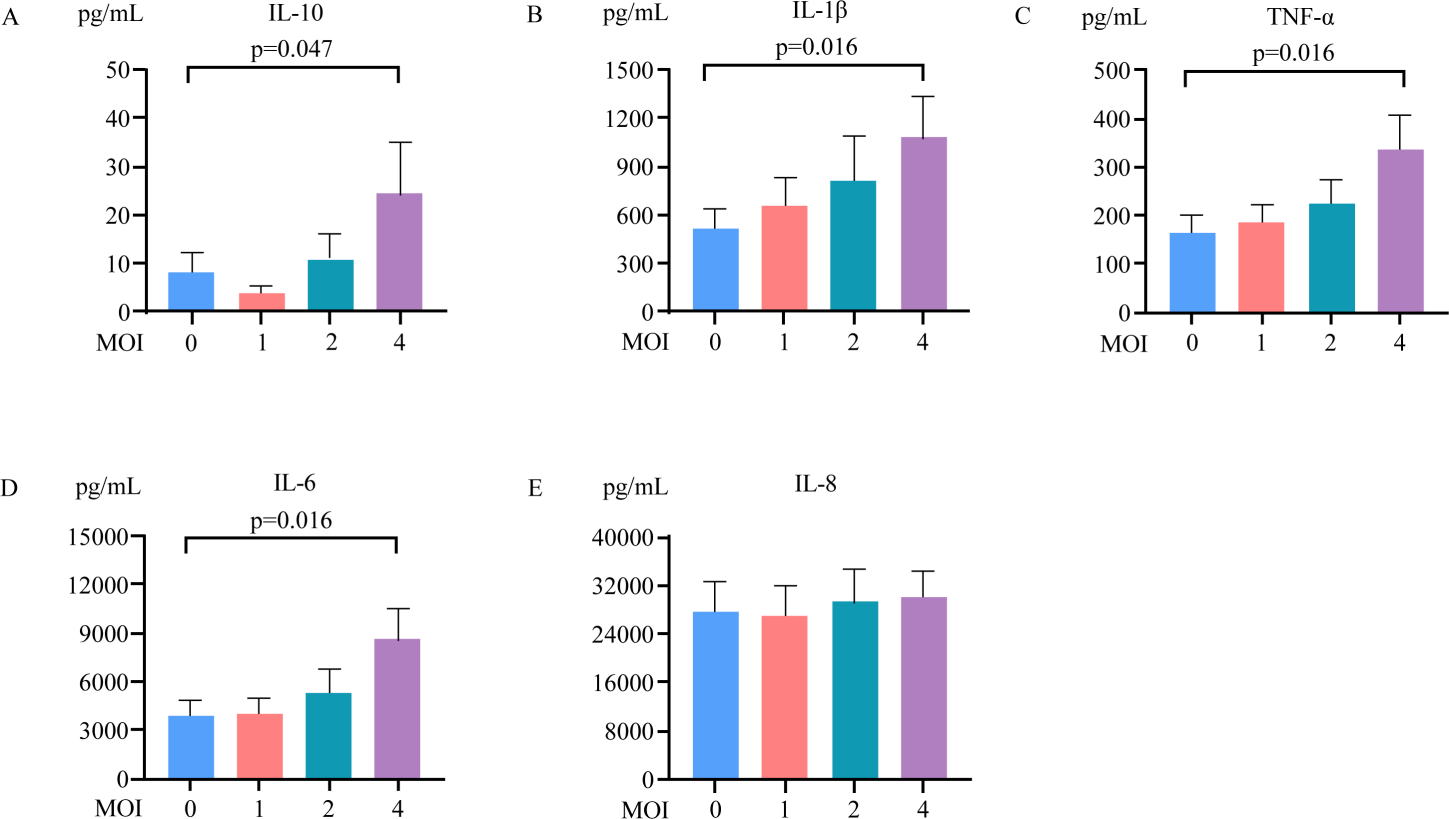
The effects of RSV infection on monocyte-derived cytokine production. Monocytes were infected with RSV at MOIs of 0, 1, 2, and 4. The cytokine levels in the culture supernatants were measured 16 h postinfection by CBA. The number of monocytes was measured at 2.5 × 10^4^ in each assay. Paired groups were compared with a two-tailed Wilcoxon matched-pairs signed-rank test.

When the levels of cytokines in the coculture supernatant of CD4^+^ T-cells and monocytes were assessed, we found that the cytokine levels were significantly altered in the presence of CD16^+^ monocytes but not in the presence of CD16^-^ monocytes following RSV infection (Fig. 3C and D). We have included a control group without RSV infection, we found that the effects of culture supernatant cytokines on Treg (Fig. S4A) and Th2 (Fig. S4B) cell proliferation were not significant changes with and without neutralizing antibodies against cytokines. Our data revealed that RSV infection of CD16^+^ monocytes increased the levels of IL-1β (*P* = 0.0078, fold change = 6.2), IL-10 (*P* = 0.0010, fold change = 7.1), TNF-α (*P* = 0.0010, fold change = 4.3), and IL-6 (*P* = 0.0061, fold change = 2.7) but not those of IL-8. To confirm that these increased cytokine levels were derived from monocytes, monocytes were infected with RSV at different MOIs. The cytokine levels in the culture supernatants were analyzed 16 h postinfection. Our data revealed that RSV infection increased the levels of IL-1β, IL-6, IL-10, and TNF-α but not the level of IL-8 in a dose-dependent manner (Fig. 4). Neutralizing antibody assays and cytokine level analysis revealed that RSV-infected CD16^+^ monocytes suppressed Treg proliferation by increasing the production of IL-1β and IL-10 and increased Th2 cell proliferation through IL-1β and TNF-α signaling.

### RSV can infect nonclassic monocytes *in vivo*

**Fig 5:**
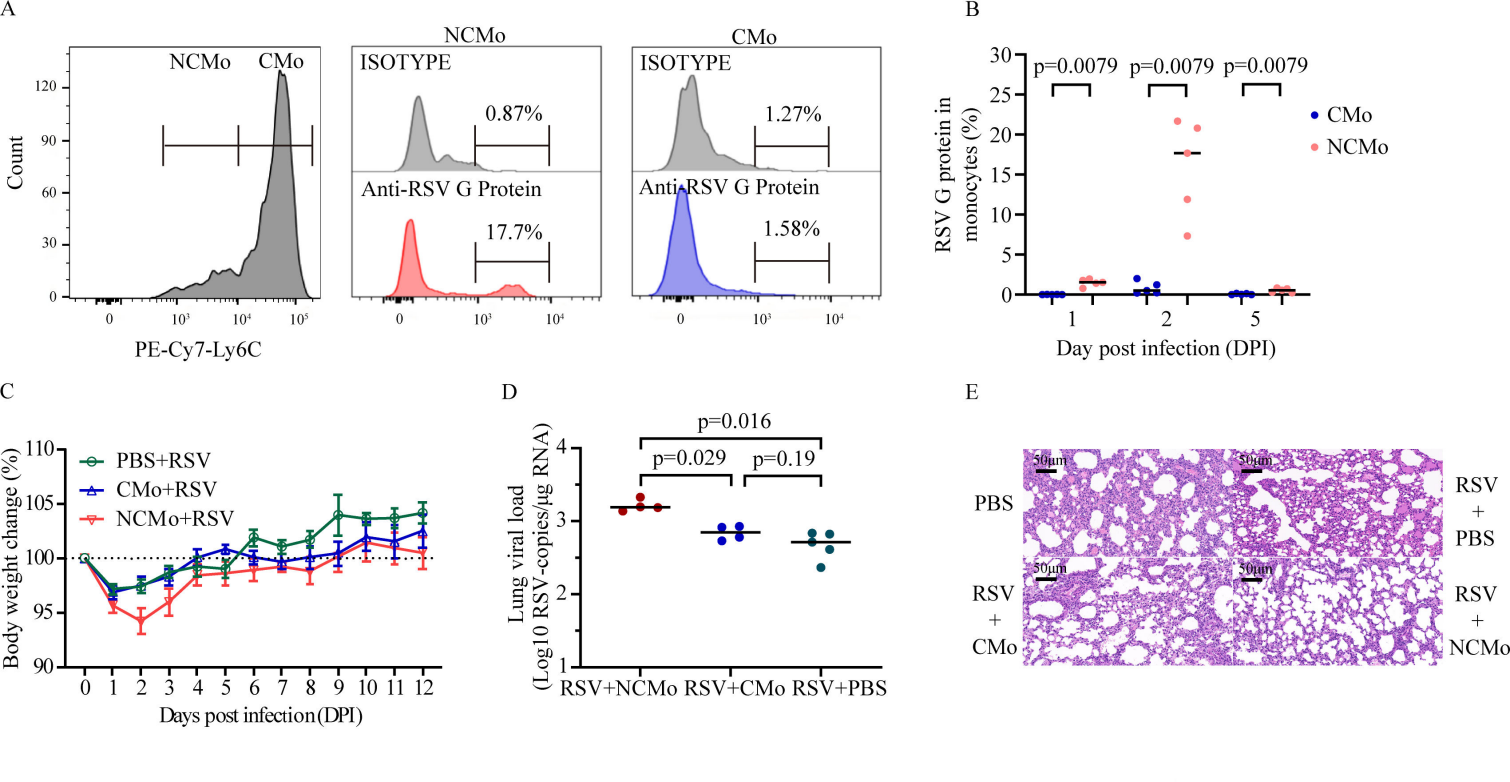
RSV infects nonclassic monocytes *in vivo*. (A) Mice were infected with RSV *via* a nasal drip, and then, lung tissues were collected 2 days postinfection to detect RSV infection in Ly6C^-^/NCMo and Ly6C^+^/CMo group mice. (B) The proportion of RSV G protein in monocytes of Ly6C^-^/NCMo and Ly6C^+^/CMo group mice was detected at different days postinfection. (C–D) Ly6C^-^/NCMo and Ly6C^+^/CMo were isolated from BALB/c mice *via* cell sorting and adoptively transferred into BALB/C *via* IV injection at the same time as RSV infection. (C) Mouse weight loss in different groups was tracked for 12 days. (D) Viral titers in the lung tissues from each group of mice analyzed by RT-qPCR. (E) H&E staining was performed to show the pathological changes in mouse lungs on day 2 postinfection. Statistical analysis was performed *via* the Mann‒Whitney U test.

Our findings indicated that RSV could effectively infect human CD16^+^ monocytes. To detect the infectivity of RSV on nonclassic monocytes *in vivo*, BALB/c mice were intranasally infected with 5 × 10^6^ PFU of RSV and sacrificed at 1, 2, and 5 days postinfection (DPI). Single-cell lung suspensions were prepared and analyzed *via* flow cytometry. All mouse monocytes were gated as CD45^+^Ly6G^-^CD11b^+^MHCII^-^ cells. Mouse classic monocytes (Ly6C^+^ and CMo) and nonclassic monocytes (Ly6C^-^ and NCMo) were further gated based on Ly6C expression (Fig. 5A). The monocytes infected with RSV were immunostained with a PE- anti-RSV G protein antibody. On day 2 postinfection, RSV-G protein-expressing cells were detected mainly in NCMo rather than in CMo (Fig. 5A and B). However, we did not detect a significant number of RSV-infected cells on day 1 or day 5 postinfection (Fig. 5B). One possible explanation for this could be that a single day is an insufficient period for RSV-infected infiltrating monocytes to express the RSV protein, and most RSV is eliminated by day 5 postinfection.

### Adoptive transfer of NCMos enhances RSV pathogenicity

These results suggest that RSV-infected NCMos can modulate the proliferation of Tregs and Th2 cells in humans. We further assessed the role of NCMos in the pathogenesis of RSV by increasing the number of NCMos and CMos through adoptive transfer. Nr4a1^-/-^ mice, which are deficient in NCMo development, were excluded from our study, as previous data indicated that Nr4a1 expression knockout could markedly increase the resistance of these mice to influenza virus infection by increasing type I interferon production (26). Similarly, methacryloyloxydecyl dihydrogen phosphate (MDP)-treated mice were not used in this study (27). Concurrently with RSV infection, BALB/c mice underwent individual adoptive transfer with NCMos (CD45^+^Ly6G^-^CD11b^+^ MHCI^-^LY6C^-^) or CMos (CD45^+^Ly6G^-^CD11b^+^ MHCI^-^LY6C^+^) through intravenous injection. Weight changes were monitored, and the lungs were subjected to histopathological examination *via* hematoxylin‒eosin (H&E) staining to analyze pathogenicity. The results revealed that RSV infection resulted in weight loss in the mice, and the Ly6C^-^/NCMo group presented the most pronounced weight loss at 2 days postinfection (*P* = 0.015 for the PBS group vs. the Ly6C^-^ group; *P* = 0.0015 for the Ly6C^+^ group vs the Ly6C^-^ group; Fig. 5C). The peak viral load was observed in the Ly6C^-^/NCMo group 2 days post-infection (Fig. 5D). H&E staining further revealed increased pulmonary interstitial edema and inflammatory cell infiltration in the Ly6C^-^/NCMo group 2 days postinfection (Fig. 5E), suggesting that NCMo administration exacerbated the severity of RSV infection in the mice.

**Fig 6:**
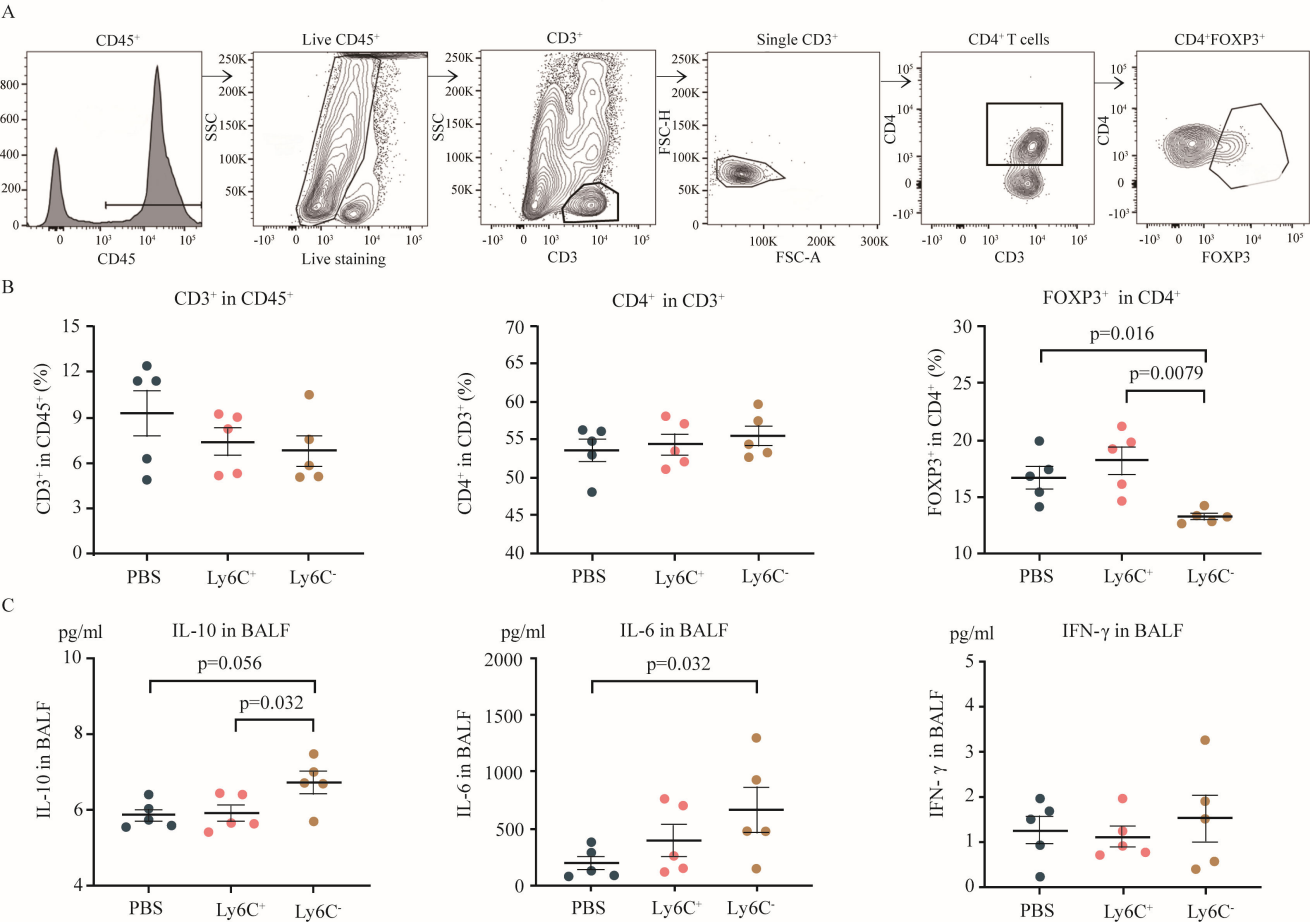
Adoptive NCMo transfer can suppress Treg cell numbers during RSV infection *in vivo*. The mice were infected with RSV along with Ly6C^+^/CMo or Ly6C-/NCMo via adoptive transfer (with PBS as a control). T-cell and T-cell subset frequencies in the lungs and cytokine levels in bronchoalveolar lavage fluid (BALF) were analyzed 2 days after RSV infection. (**A**) Gating strategy for Tregs in mouse lungs. (**B**) The frequencies of total T cells, CD4^+^ T cells, and Tregs in the lungs were analyzed by flow cytometry. (**C**) The levels of IL-10, Il-6, and IFN-γ in the BALF were analyzed by CBA. Multiple comparisons were performed via the Kruskal‒Wallis test.

Our *in vitro* experimental data suggest that RSV infection of human CD16^+^ monocytes result in a decrease in Treg frequency. To analyze the impact of RSV-infected NCMos on Tregs *in vivo,* the frequencies of total CD4^+^ T cells and Tregs in the lungs were determined by flow cytometry 2 days after RSV infection (Fig. 6A). The data revealed that the number of Treg cells in the Ly6C^-^/NCMo group was lower than that in the PBS group (*P* = 0.016) and the Ly6C^+^/CMo group (*P* = 0.0079; Fig. 6B). Nevertheless, adoptive transfer of NCMos had no effect on the total quantity of CD4^+^ T-cells (Fig. 6B). Our findings suggest that the impact of NCMos is predominantly focused on Tregs. NCMos infected with RSV increase RSV pathogenicity by reducing the quantity of Tregs. The levels of various inflammatory cytokines in bronchoalveolar lavage fluid (BALF) revealed that the IL-10 concentration in Ly6C^-^/NCMo group mice was greater than that in Ly6C^+^/CMo group mice (*P* = 0.032, Fig. 6C). The IL-6 levels in the Ly6C^-^/NCMo group were greater than those in the PBS group (*P* = 0.032, Fig. 6C). We did not detect significant differences in the IFN-γ, TNF-α, or MCP-1 concentrations among the mice in the Ly6C^-^/NCMo group, Ly6C^+^/CMo group, or PBS group (Fig. 6C; Fig. S5). Combining data from human blood cells *in vitro* and mice *in vivo*, we found that the novel NCMo/IL-10/Treg axis may be critical for the pathogenesis of RSV in both humans and mice.

## DISCUSSION

In this study, we demonstrated that RSV targets CD16^+^ nonclassic monocytes, thereby perturbing the immune balance of CD4^+^ T-cell subsets, namely, by suppressing the number of Tregs and increasing the number of Th2 cells. Our results further revealed that the effects on RSV-infected CD16^+^ monocytes were mediated through IL-1β and IL-10. The *in vivo* data from the mice confirmed the preference for NCMos by RSV and identified NCMos as a pathogenicity factor during RSV infection, which supported our *in vitro* findings that RSV-infected NCMos suppressed the increase in Treg numbers *via* IL-10 signaling. Overall, we identified the RSV-nonclassic monocyte/IL-1β/IL-10/CD4^+^ T-cell subset balance as a novel RSV pathogenesis pathway.

Our data showed that RSV-infected NCMos can promote RSV pathogenicity. Although many reports have shown that CMo and NCMo number/frequency, gene expression profiles, and activity are altered in various infectious diseases, including RSV (28–31), few studies have focused on determining the mechanism by which monocyte subsets contribute to the pathogenesis of diseases. However, these studies still revealed that microbes can increase their pathogenicity by hijacking CD14^+^CD16^+^ monocytes. For example, Dengue virus infection induces CD14^+^CD16^+^ monocyte differentiation, which can stimulate plasmablast B-cell differentiation and IgG/IgM secretion. CD14^+^CD16^+^ monocytes may help neutralize Dengue virus infection but enhance disease upon infection with another, secondary serotype, Dengue virus (32). Understanding the mechanism by which pathogens hijack monocyte subsets not only expands our knowledge of pathogen and immune system interactions but also provides potential therapeutic targets for disease. In RSV infection, CD16^+^ monocyte-targeted therapy may alleviate disease severity and reduce the health-care service burden by decreasing hospitalization.

Our results suggest that NCMos increase RSV pathogenicity through Treg and Th2 cell subsets. Tregs can prevent exacerbation of the immune response to RSV by suppressing T-cell proliferation and cytokine production and accelerate RSV clearance by facilitating RSV-specific CD8^+^ T-cell recruitment at the early stage (33, 34). The immune response to RSV in mice is Th1-dominant, but the Th2 response can still exacerbate disease severity by increasing mucus production and airway hyperreactivity; these can be achieved by blocking Th2-associated cytokines, including IL-13, and depleting cell subsets that inhibit the Th2 response, including NK cells and Tregs (35). On the basis of these findings, we speculate that NCMos increase RSV disease severity by exacerbating inflammation, delaying virus clearance, and increasing airway resistance.

A previous study has shown that human CD16^+^ monocytes induce Th1 cell expansion under basal conditions (17). However, in the present study, we observed that CD16^+^ monocytes induce Th2 cell expansion under RSV infection conditions through IL-1β and IL-10. This discrepancy is likely attributable to RSV infection inducing robust inflammatory cytokine production from CD16^+^ monocyte. The functions of monocyte subsets are plastic and depend on the context of inflammation and immune response. For example, CD16^+^CD14^dim^ monocytes in human, similar to CX3CR1^high^ LY6C^-^ monocytes in mice, continuously patrol the luminal side of vascular endothelium to remove damaged cell and debris under steady-state conditions (36). However, this monocyte subset also can promote intravascular neutrophils activation, leading to glomerular injury during nephritis (37). Studies focusing on changes in the activity of monocyte subsets during RSV infection will help improve the understanding of RSV pathology. Studies have demonstrated that CD16^+^ monocytes play a significant role in the pathogenesis and severity of COVID-19 (38, 39).

In the present study, we found that RSV-infected NCMos inhibited the increase in Treg numbers through IL-1β and IL-10 signaling. As shown by our data and other reports, RSV infection can induce monocyte-derived IL-1β production. In addition, our results are also supported by reports indicating that IL-1β inhibits Treg differentiation and activity and converts Tregs into Th17 cells through the induction of alternative splicing of Foxp3 and HIF-1α expression (40). However, it is surprising that IL-10 inhibits the increase in Treg numbers in the presence of RSV infection because IL-10 has been shown to facilitate Treg differentiation and function in most situations. One potential mechanism by which IL-10 inhibits Treg proliferation is that IL-10 can inhibit T-cell production of IL-2 (41), a key cytokine for Treg proliferation, and can dampen IL-2 activity indirectly through antigen-presenting cells. This potential is supported by reduced IL-2 production and by the limited response to IL-2 in RSV-infected infants (42). Another possibility is the context-dependent pleiotropic effects of IL-10. As a well-known anti-inflammatory cytokine, IL-10 can potentiate systemic inflammation in humans with experimental endotoxemia and increase NK-derived IFN-γ production in psoriatic patients (43, 44). A study reporting this mechanism also revealed that IFN-γ signaling could reprogram IL-10 activity to induce proinflammatory STAT1 activation instead of STAT3 activation (the originally identified IL-10 signaling pathway) (45).

Our data demonstrate that RSV-infected CD16^+^ monocytes produce IL-1β, which inhibits Treg and increases Th2 proliferation. In human monocytes, IL-1β production is both subset- and cell signaling-dependent. CD14^+^CD16^+^ monocytes secrete high levels of IL-1β in the presence of either LPS or TLR7/8, a viral ssRNA agonist (46). However, CD14^+^CD16^-^ monocytes and CD14^dim^CD16^+^ monocytes are sensitive to only LPS or TLR7/8 stimulation, respectively. During RSV infection, multiple types of TLR signaling can be activated, including triggering of TLR4 through the RSV F protein and triggering of TLR7/8 through the presence of ssRNA (47). In addition, RSV has been shown to induce IL-1β production by activating the NLRP3/ASC inflammasome through TLR2, which is also expressed at relatively high levels in CD14^+^CD16^+^ monocytes (48). One study revealed that the difference in the F protein expression between RSV strains was associated with IL-1β production, although the mechanism is unclear (46). In summary, the specific innate immune signaling pathway that triggers IL-1β production in distinct monocyte subsets during RSV infection needs to be elucidated through further studies. Because CD14^+^CD16^+^ monocytes are sensitive to multiple signaling pathways, they produce greater amounts of IL-1β after activation, and their number increases during RSV infection, we speculate that CD14^+^CD16^+^ monocytes are the major source of IL-1β during RSV infection.

Our data suggest that NCMos are prone to RSV infection in both humans and mice, although the exact mechanism of action remains to be elucidated. The RSV G protein contains a CX3C motif, which binds to CX3CR1, the receptor for fractalkine (49, 50). CX3CR1 is highly expressed on NCMos but not on CMos (51). This finding suggests that CX3CR1 is a potential candidate receptor for RSV-infecting NCMos (52). In addition to the RSV G protein, the RSV F protein is also critical for viral infectivity (53). Studies revealed that the F protein interacts with several proteins to facilitate viral entry, including intercellular adhesion molecule 1 (ICAM1) (54), epidermal growth factor receptor (EGFR) (55, 56) and nucleolin (57, 58). We will identify these candidates by analyzing their expression levels on NCMos/CMos and blocking interactions with neutralizing antibodies and/or gene expression knockout in future studies.

It has been reported that the elevated IL-6 levels in the airway and blood are indicative of severe RSV infection (59). In this study, we found that RSV infection of total monocytes or CD16^+^ monocytes increased the levels of IL-6. The role and mechanism of IL-6 in RSV infection needs to be investigated in future studies.

There are several limitations in this study. First, the antigenic specificity of Tregs and their function were not evaluated in this study. Second, we did not conduct an analysis of the mechanisms through which RSV enter monocytes in this study.

In conclusion, we identified an RSV-nonclassic monocyte/IL-1β/IL10/CD4^+^ T-cell subset that balances the pathogenesis pathway, which connects monocyte heterogenicity with RSV pathogenesis, and elucidated a new mechanism by which RSV skews the CD4^+^ T-cell balance during RSV infection. The novel findings of the present study provide potential therapeutic targets for RSV infection and RSV-associated complications.

## MATERIALS AND METHODS

### Human blood samples

Fresh leukocyte-enriched peripheral blood samples were obtained from healthy donors without any identifiers at the Beijing Red Cross Blood Center. This study was approved by the Institutional Review Boards of the Institute of Pathogen Biology, Chinese Academy of Medical Sciences (IPB-2018–3).

### Viruses and cells

The respiratory syncytial virus (RSV) A2 strain (VR-1540) was purchased from the American Type Culture Collection (ATCC; Manassas, VA, USA). H3N2 was provided by the Institute of Laboratory Animal Sciences, CAMS & PUMC. The human laryngeal carcinoma cell line HEp-2 (CCL-23) was purchased from the ATCC and maintained in Dulbecco’s modified Eagle’s medium (DMEM, Invitrogen, Carlsbad, CA, USA) supplemented with 10% fetal bovine serum (HyClone, Logan, UT, USA), 100 U/mL penicillin and 100 mg/mL streptomycin at 37°C with 5% CO_2_.

### Cell isolation and purification

Peripheral blood mononuclear cells (PBMCs) were separated from leukocyte-enriched blood samples using Ficoll–Paque PLUS (GE Healthcare, Chicago, IL, USA) according to the manufacturer’s instructions. Total CD4^+^ T cells and monocytes were purified via a CD4^+^ T-cell isolation kit and CD14 microbeads (Miltenyi Biotec, Aubum, CA), respectively (both purities > 95%). CD16^+^ and CD16^-^ monocytes were purified from CD14 monocytes *via* fluorescence-activated cell sorting and incubated with PE-Cy7-anti-human CD14 and APC-anti-human CD16 antibodies (BD Biosciences, NY, USA) (both purities > 99%).

### RSV/H3N2 adsorption and infection assays

Purified human monocytes were resuspended at a final concentration of 1 × 10^6^ /mL and infected with RSV/H3N2 at an MOI of 16 on ice for 2 h to investigate the adsorption of RSV/H3N2 by monocytes. For the infection assay, monocytes were infected with RSV/H3N2 at an MOI of 4 at 37°C for 2 h and washed with loading buffer (LB) containing 1× D PBS (Thermo Fisher Scientific, NY, USA) with 2 mM ethylenediaminetetraacetic acid (EDTA, Thermo Fisher Scientific) and 0.05% BSA (Sigma, St. Louis, MO, USA). Then, the monocytes were maintained in R10 medium (RPMI 1640 medium [Thermo Fisher Scientific] supplemented with 10% fetal bovine serum [FBS, HyClone], 2 mM glutamine [Invitrogen], 5 mM HEPES [Thermo Fisher Scientific], and 0.2 mM sodium pyruvate) for 16 h. The monocytes that had adsorbed or were infected with RSV/H3N2 were collected. After incubation with Foxp3/transcription factor fixation permeabilization concentrate and diluent (Thermo Fisher Scientific) at 4°C for 45 min, the cells were incubated with APC- anti-RSV-G protein (Abcam, Cambridge, UK) at 4°C for 45 min. All the samples were acquired on an LSRFortessa flow cytometer and analyzed *via* FlowJo software (BD Biosciences).

### CD4^+^ T-cell and monocyte coculture

After being resuspended at a final concentration of 1 × 10^6^ /mL, the purified CD4^+^ T cells were stained with CellTrace CFSE staining solution (Invitrogen) for 20 min at room temperature. CFSE-labeled CD4^+^ T cells were resuspended in R10 medium and mixed with autologous total monocytes infected with RSV/H3N2 at a ratio of 2:1, after which a soluble anti-CD3 antibody (BD Biosciences) was added. The mixture was subsequently incubated at 37°C for 7 days. Alternatively, CFSE-labeled CD4^+^ T cells and purified CD14^+^CD16^-^ cells (2:1 ratio) were cocultured together with or without CD16^+^ monocytes (CD14^+^CD16^-^/CD16^+^ monocyte ratio of 2:1) and an anti-CD3 antibody for 7 days.

### Antibody blocking assays

To detect cytokines that affect Treg and Th2 proliferation, we performed antibody blocking studies. Neutralizing antibodies against human IL-6, human IL-10, human TNF, human IL-13, human IL-1β, human IL-27, and isotype-matched controls were added to CFSE-labeled purified CD4^+^ T-cells (1 × 10^6^ cells/mL) and then cocultured with autologous total monocytes or subsets infected with RSV/H3N2 for 7 days.

### Intracellular and surface expression analyses

On day 7 after coculturing CD4^+^ T cells and monocytes, the cells were harvested. Surface staining was performed with APC-Cy7-anti-human CD3, Alexa Fluor 700-anti-human CD4, and PerCP-Cy5.5-anti-human CD8 antibodies. After incubation with Foxp3 fixation/permeabilization solution, the cells were immunostained with PE-Cy7-anti-human Foxp3. All samples were acquired on an LSRFortessa flow cytometer and analyzed *via* FlowJo software. CD4^+^ T cells that had divided were defined as the CFSE^lo^CD3^+^CD4^+^ population. Tregs were identified as Foxp3^hi^CD4^+^ T cells.

On day 7 after coculturing CD4^+^ T-cells and monocytes, the cells were stimulated with PMA (Sigma), ionomycin (Sigma), brefeldin A (GolgiPlug, Biolegend, San Diego, CA), and monensin (GolgiStop, Biolegend) at 37°C for 5 h. After harvesting, surface staining was performed with APC-Cy7- anti-human CD3, Alexa Fluor 700-anti-human CD4, and PerCP-Cy5.5-anti-human CD8 antibodies. After incubation with the Foxp3 fixation/permeabilization solution, the cells were immunostained with APC-anti-human IFN-γ, PE-anti-human IL-4, or PE-Cy7-anti-human IL17A. All samples were acquired on an LSRFortessa flow cytometer and analyzed *via* FlowJo software. CD4^+^ T-cells that had divided were defined as the CFSE^lo^CD3^+^CD4^+^ population. Th1 cells were identified as IFN-γ^hi^ CD4^+^ T-cells, Th2 cells were identified as IL-4^hi^ CD4^+^ T-cells, and Th17 cells were identified as IL-17A^hi^ CD4^+^ T-cells.

### Animal experiments

All animal experiments were approved by the Animal Ethics and Experimentation Committee of the Institute of Pathogen Biology of the Chinese Academy of Medical Sciences (2018S07). Female BALB/c mice aged 6–8 weeks were purchased from Beijing HFK Bioscience Company (Beijing, China) and maintained in an animal biosafety level 2 (ABSL-2) facility at the Beijing E-Town Biopharm Generic Technology Platform. NCMos (CD45^+^Ly6G^-^CD11b^+^MHCII^-^Ly6C^-^) and CMos (CD45^+^Ly6G^-^CD11b^+^MHCII^-^Ly6C^+^) were isolated from the blood and spleens of BALB/c mice *via* a FACSAria II Sorter (BD Biosciences). Female BALB/c mice were randomly assigned to the Ly6C^+^/CMo group, Ly6C^-^/NCMo group, or PBS group. Simultaneously, either Ly6C^+^ (Ly6C^+^/CMo group, *n* = 25) or Ly6C^-^ (Ly6C^-^/NCMo group, *n* = 25) monocytes were injected into each mouse *via* the tail vein, and the PBS group received an equal volume of PBS. Five hours later, the mice were anesthetized with tribromoethanol (0.25  mg/g mouse) and treated intranasal with 50 µL of RSV (5 × 10^6^ pfu.). Ten mice per group were observed for 12 days, and their weight loss was tracked. Fifteen mice per group were observed for 5 days and were euthanized on days 1, 2, or 5. The BALF was harvested to determine cytokine levels. For histopathological analysis, the lungs were harvested and fixed with 4% paraformaldehyde (PFA). Lung samples were embedded in paraffin and examined *via* H&E histopathological examination. To assess changes in the CD4^+^ T-cell subsets, the lungs were digested in enzymatic digestion buffer supplemented with 4 mg/mL collagenase IV (Sigma), 0.4 mg/mL collagenase/dispase (Roche, Germany), 30 U/mL DNase (Promega, WI, USA), and 0.5% BSA in HBSS (Thermo Fisher Scientific) at 37°C and 100 rpm for 45 min. The lung cells were resuspended in LB buffer. Dead cells were labeled with BD Horizon Fixable Viability Stain 510 (BD Biosciences). Surface staining was performed for 20 min at 4°C using APC-Cy7-anti-mouse-CD45, BV421-anti-mouse-Ly6G, PerCP-Cy5.5-anti-mouse-CD11b, PE-Cy7-anti-mouse-Ly6C, BV650-anti-mouse MHC class II (I-A/I-E), and Alexa Fluor 700-anti-mouse-CD11c antibodies. After being fixed and permeabilized with Foxp3 fixation/permeabilization solution at 4°C for 45 min, the cells were washed with permeabilization buffer for two times and incubated with PE-anti-RSV G at 4°C for 45 min. After being washed with permeabilization buffer, the cells were resuspended in 100 µL PBS for analysis. All samples were acquired on an LSRFortessa flow cytometer and analyzed *via* FlowJo software.

### Determination of RSV titer from lung tissues

The lung tissues were collected and ground with a tissue homogenizer. Then, the total RNA was extracted by Trizol from homogenates. The viral RNA sample (5 µL/25 ng) or RNA reference standard (10^7^ to 10^0^) was mixed with nucleic acid amplification reaction solution mix (4 µL), RSV specific primers (0.2 µL/0.5 µM,) and probes (0.2 µL/0.25 µM) targeting the N gene and water (9.4 µL). The real-time RT-PCR assay was performed on a CFX96 Touch Real-Time PCR Detection System (BIO-RAD, Hercules, CA, USA) with cycling conditions of 50°C for 15 min, 95°C for 10 min, followed by 40 cycles of 95°C for 15 s, 60°C for 1 min.

### Cytokine secretion assays

Inflammatory cytokines in monocyte and CD4^+^ T-cell coculture supernatants and mouse BALF samples were detected *via* a BD Cytometric Bead Array (CBA) Human or Mouse Inflammatory Cytokines Kit (BD Biosciences, NY, USA) following the manufacturer’s instructions.

### H&E staining

Lungs from the Ly6C^+^/CMo group, Ly6C^-^/NCMo group, and PBS group were collected. After fixation with 4% PFA (Boster, CA, USA), the lungs were embedded in paraffin wax. Lung sections (5 mm thick) were stained with H&E and examined for histopathological changes.

### Statistical analysis

Single comparisons between other metrics were performed *via* the Mann‒Whitney U test. Multiple comparisons were performed *via* the Kruskal‒Wallis test. Paired groups were compared with a two-tailed Wilcoxon matched-pairs signed-rank test. Multiple paired comparisons were performed using a Friedman test. A two-sided *P* < 0.05 was considered statistically significant. All the statistical analyses were conducted *via* GraphPad Prism 10.2 software.

## SUPPLEMENTAL MATERIAL

Supplemental table and figures spectrum.02073-24-s0001.docx Table S1; Fig. S1 to S4.

ASM does not own the copyrights to Supplemental Material that may be linked to, or accessed through, an article. The authors have granted ASM a non-exclusive, world-wide license to publish the Supplemental Material files. Please contact the corresponding author directly for reuse.
