# Supplementary material for: Infection of nonclassic monocytes by respiratory syncytial virus induces an imbalance in the CD4+ T-cell subset response
Source: Microbiol Spectr. 2024 Dec 10;13(1):e02073-24. doi: 10.1128/spectrum.02073-24 (PMC11705840; doi:10.1128/spectrum.02073-24)
Supplement: Supplemental table and figures — Table S1; Fig. S1 to S4. [file spectrum.02073-24-s0001.docx]

**Infection of nonclassic monocytes by respiratory syncytial virus induces an imbalance in the CD4^+^ T-cell subset response**

Lianlian Han ^#1^, Danyang Li ^#1^, Conghui Wang ^1^, Lili Ren^1,2,3^, Li Guo* ^1,2,3^, Jianwei Wang* ^1,3^

**Supplemental table 1. Reagents used in this study**

| **Reagent** | **Information** | **Source** | **Identifier** |
| --- | --- | --- | --- |
| **Antibodies** | **Clone** |  |  |
| PE-cy7-anti-human CD14 | MφP9 | BD Biosciences | 562698 |
| APC-anti-human CD16 | B73.1 | BD Biosciences | 561304 |
| anti-RSV-G protein | RSV133 | Abcam | ab94966 |
| Purified NA/LE Mouse Anti-Human CD3 | HIT3a | BD Biosciences | 555336 |
| Purified NA/LE Rat Anti-Human IL-6 | MQ2-13A5 | BD Biosciences | 554541 |
| Purified NA/LE Rat Anti-Human and viral IL-10 | JES3-9D7 | BD Biosciences | 554495 |
| Purified NA/LE Mouse Anti-Human TNF | MABTNF-A5 | BD Biosciences | 552467 |
| Purified Mouse Anti-Human IL-1β | AS10 | BD Biosciences | 550007 |
| Human IL-13 Antibody | 32116 | R&D Systems | MAB213100 |
| Human IL-27 Antibody | NA | R&D Systems | AF2526 |
| Alexa Flour 700-anti- human CD4 | SK3 | BD Biosciences | 566392 |
| APC-cy7-anti- human CD3 | SK7 | BD Biosciences | 557832 |
| Percy5.5-anti-human CD8 | RPA-T8 | BD Biosciences | 560662 |
| PE-cy7-anti-human Foxp3 | PCH101 | Invitrogen | 25-4776-42 |
| PE-anti-human IL-4 | 8D4-8 | BD Biosciences | 562046 |
| PE-cy7-anti-human IL17A | eBio64DEC17 | Invitrogen | 25-7179-41 |
| APC-anti-human IFN-γ | B27 | BD Biosciences | 562017 |
| APC-Cy7-anti-mouse-CD45 | 30-F11 | BD Biosciences | 557659 |
| BV421-anti-mouse-Ly6G | 1A8 | BD Biosciences | 562737 |
| PerCP-Cy5.5-anti-mouse-CD11b | M1/70 | BD Biosciences | 550993 |
| PE-Cy7-anti-mouse-Ly6C | HK1.4.rMAb | BD Biosciences | 569443 |
| BV650-anti-mouse MHC class II | 2G9 | BD Biosciences | 743873 |
| Alexa Fluor 700-anti-mouse-CD11c | N418 | Invitrogen | 56-0114-82 |
| Human Immunoglobulin Isotyping Cytometric Bead Array (CBA) Kit | NA | BD Biosciences | 551811 |
| Mouse Immunoglobulin Isotyping Cytometric Bead Array (CBA) Kit | NA | BD Biosciences | 550026 |
|  |  |  |  |
| Carboxyfluorescein diacetate succinimidyl ester (CFSE) | NA | Invitrogen | C1157 |
|  |  |  |  |
| **Live/Dead buffer** |  |  |  |
| BD Horizon Fixable Viability Stain 510 | NA | BD Biosciences | 564406 |
|  |  |  |  |
| **Stimulates** | **Clone** |  |  |
| PMA | NA | Sigma | P8139 |
| Ionomycin | NA | Sigma | I3909 |
| Brefeldin A Solution (1,000X) | NA | Biolegend | 420601 |
| Monensin Solution (1,000X) | NA | Biolegend | 420701 |
|  |  |  |  |
| Collagenase IV | NA | Sigma | C4-BIOC |
| Collagenase/dispase | NA | Roche | 10269638001 |
| RQ1 RNase-Free DNase | NA | Promega | M6101 |
|  |  |  |  |
| Red cell lysis solution | NA | TBD | NH4CL2009 |
| Foxp3/Transcription Factor Fixation Permeabilization Concentrate and Diluent | NA | Invitrogen | 00-5521-00 |
| Foxp3 fixation/permeabilization solution | NA | Invitrogen | 00-8333-56 |
| 4% PFA | NA | Boster | AR1068 |
|  |  |  |  |
| Carboxyfluorescein diacetate succinimidyl ester (CFSE) | NA | Invitrogen | C1157 |
| 1× D-PBS | NA | Gibco | 14190144 |
| Ethylenediaminetetraacetic acid (EDTA) | NA | Gibco | 15400054 |
| BSA | NA | Sigma | A1933-100G |
| RPMI1640 | NA | Gibco | 11879020 |
| Fetal bovine serum | NA | Gibco | A5670701 |
| Dulbecco’s Modified Eagle’s Medium (DMEM) | NA | Invitrogen | 41090036 |
| Penicillin, Streptomycin, Glutamine (100X) | NA | Invitrogen | 15070063 |
| HEPES | NA | Gibco | 15630130 |
| Sodium Pyruvate | NA | Invitrogen | 11360070 |

**Supplemental Figure 1. RSV adsorbs and infects monocytes.**


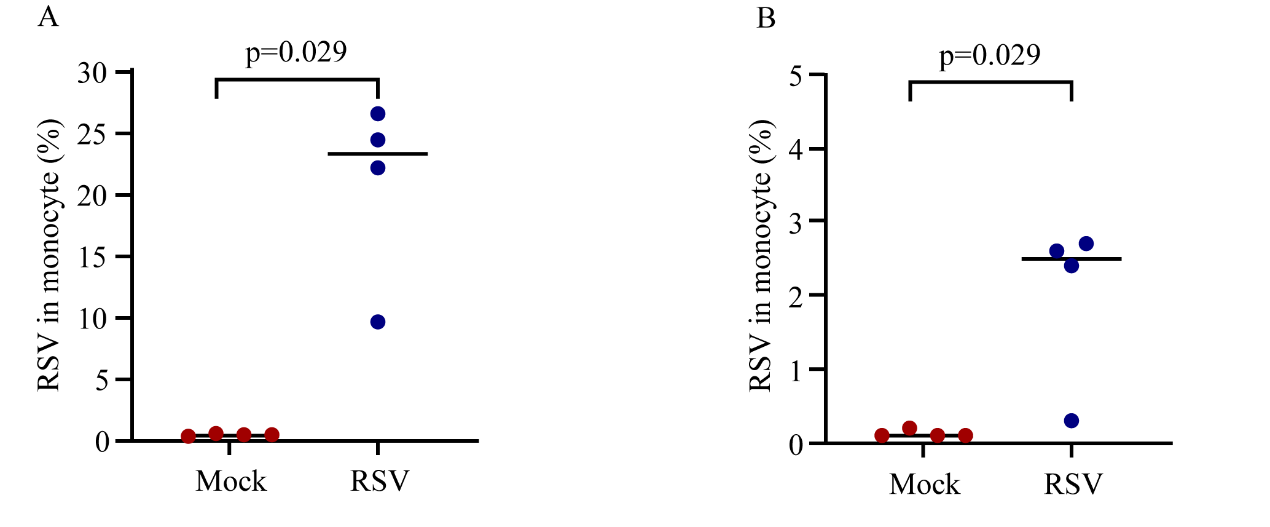


Monocytes were infected with or without RSV at 4 °C (adsorption) or 37 °C (infection). The adsorption (A) and infection (B) of monocytes with RSV were determined by flow cytometry.

**Supplemental Figure 2. H3N2-infected monocyte.**


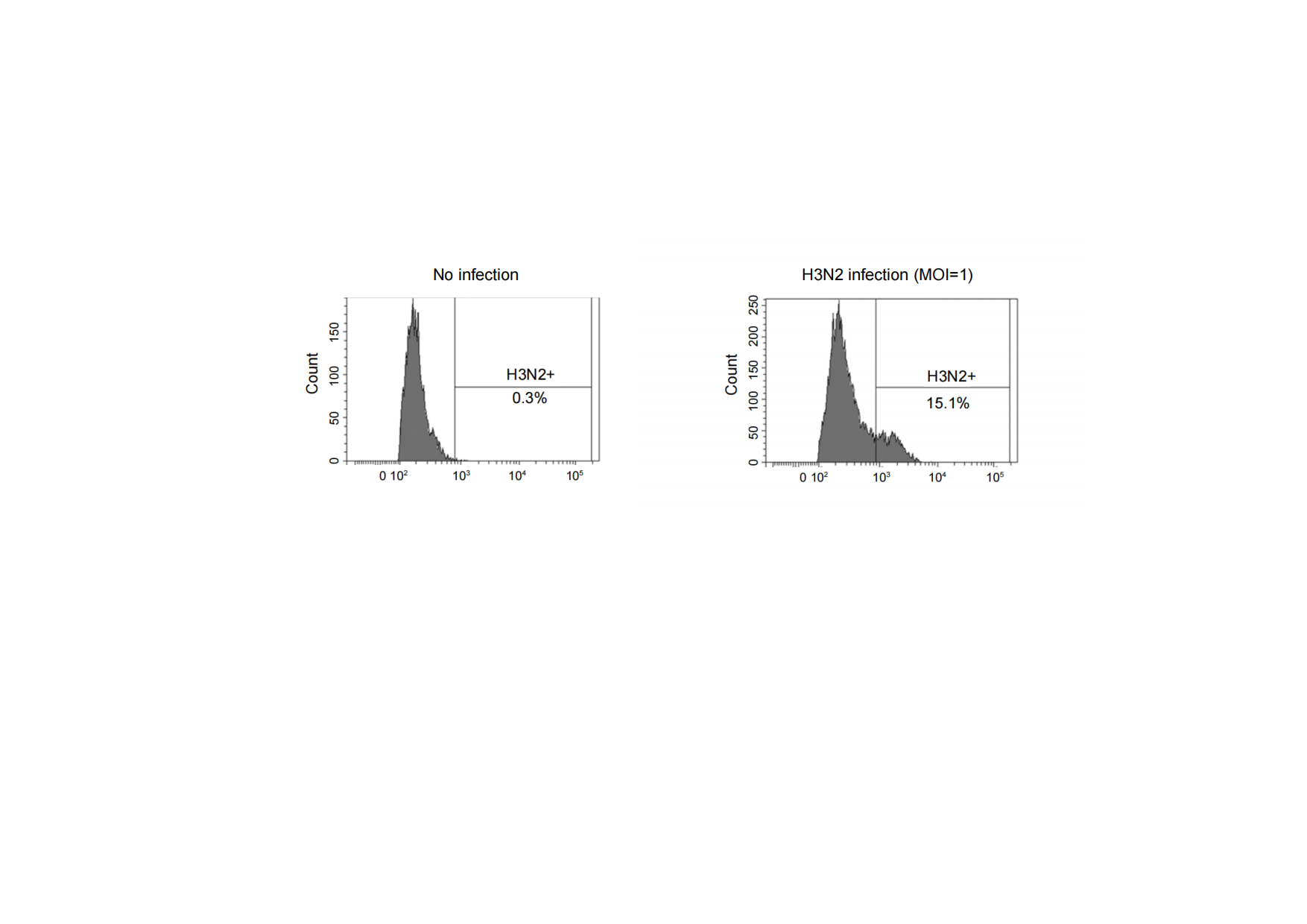


Monocytes were infected with or without H3N2 at 37℃ (infection). The infection of monocytes without (A) and with H3N2 (B) were determined using APC-conjugated anti-H3N2 NP antibody by flow cytometry.

**Supplemental Figure 3. RSV-infected HEp-2 cells.**


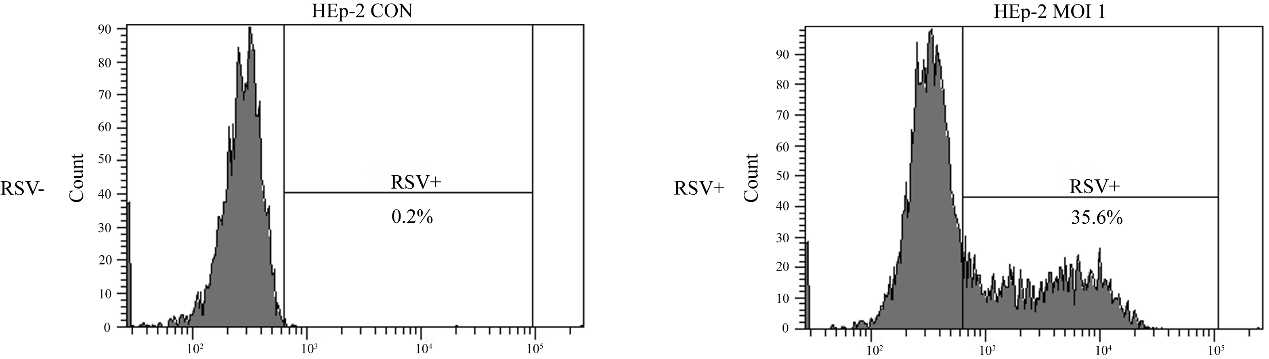


HEp-2 cells were infected with RSV for 16 h. The frequency of RSV-infected HEp-2 cells was detected via intracellular staining with an APC-conjugated anti-RSV G protein antibody and analyzed via flow cytometry.

**Supplemental Figure 4. Cytokine effects on Treg and Th2 cell proliferation without RSV infection.**


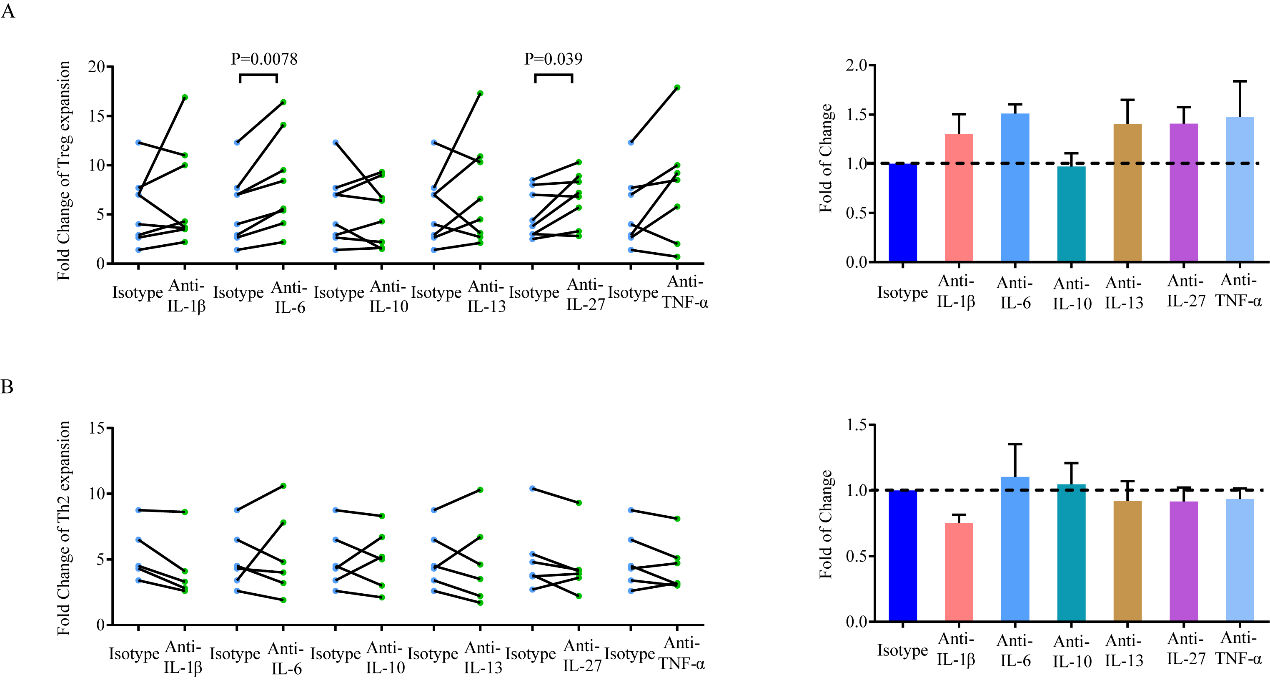


Monocytes and CD4^+^ T-cells were cocultured. The effects of culture supernatant cytokines on Treg (**A**) and Th2 (**B**) cell proliferation were compared between the groups treated with and without neutralizing antibodies against cytokines.

**Supplemental Figure 5. The levels of TNF-α and MCP-1 in bronchoalveolar lavage fluid.**


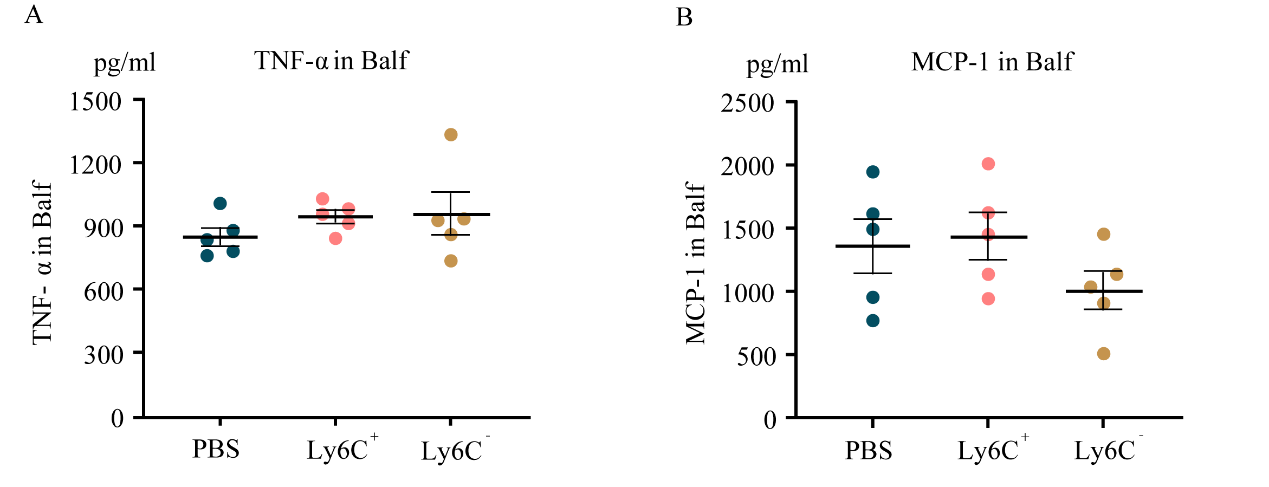


The mice were infected with RSV along with Ly6C^+^/CMo or Ly6C^-^/NCMo via adoptive transfer (with PBS as a control). The levels of TNF-α and MCP-1 in the bronchoalveolar lavage fluid (BALF) were analyzed at 2 days post-RSV infection via CBA.
